# The protective role of Wnt3a in peroxynitrite-induced damage of cochlear hair cells in vitro

**DOI:** 10.1016/j.bjorl.2023.101278

**Published:** 2023-06-03

**Authors:** Fengyun Cui, Zhimin Cao, Qianru Zhang, Zhixin Cao

**Affiliations:** a Shandong Provincial Hospital Affiliated to Shandong First Medical University, Department of Pathology, Shandong Province, China; b Gao Tang People’s Hospital Affiliated to Jining Medical University, Emergency Department, Shandong Province, China

**Keywords:** Cochlear hair cells, Wnt3a, Hearing loss, Peroxynitrite

## Abstract

**Objective:**

To investigate the effect of peroxynitrite on the cultured cochlear hair cells of C57BL/6 P3 mice in vitro as well as the role of Wnt3a, as an activator of the canonical Wnt signaling pathway, underlying the action of such an oxidative stress.

**Methods:**

The in vitro primary cultured cochlear hair cells were subjected to l00 μM peroxynitrite and l00 μM peroxynitrite +25 ng/mL Wnt3a for 24 h, the cell survival and morphological changes were examined by immunofluorescence and transmission electron microscopy.

**Results:**

The number of surviving hair cells was significantly reduced in the 100 μM peroxynitrite group, while it was significantly higher in the Wnt3a + peroxynitrite treated group compared with the peroxynitrite treated group. The transmission electron microscopy showed that exposure to peroxynitrite induced a dramatic decrease in the number of mitochondria and severely disrupted mitochondrial ultrastructure, while Wnt3a clearly diminished the disruption of mitochondrial structure and preserved a higher number of mitochondria.

**Conclusion:**

These results indicated that peroxynitrite could cause oxidative damage to the cochlear hair cells, and low concentrations of Wnt3a has a protective effect against oxidative damage.

**Level of evidence:**

Level 2.

## Highlights

• Peroxynitrite could induce Hair Cells (HCs) damage in vitro on mouse cochlear.
• Peroxynitrite eventuated HCs loss in a basal-apical gradient manner.
• Outer HCs were more vulnerable to peroxynitrite injury than inner HCs.
• Peroxynitrite could cause mitochondria oxidative damage to HCs.
• Low concentrations of Wnt3a have a protective effect on the oxidative damage of HCs.

## Introduction

According to the latest statistics from the World Health Organization, over 5% of the world’s population, that is, more than 360 million people suffers from disabling hearing loss across the globe.^1^ Deafness has become a major concern in the social and professional lives of the patients.^2^ Cochlear Hair Cells (HCs) are the sensory cells of the auditory system, which are irreversible and not replaced spontaneously.^3^ A large number of studies have found that oxidative stress has been implicated as a cause of hair cell damage under pathological conditions such as hypoxia, aging, noise trauma, and ototoxic drugs.^4^, ^5^, ^6^, ^7^ However, long-term chronic or excessive oxidative stress can lead to irreversible damage of inner ear HCs and eventually lead to permanent Sensorineural Hearing Loss (SNHL), therefore, it is essential to study oxidative damage to cochlear hair cells for the prevention and treatment of SNHL.

Wnt3a is an agonist of the classical Wnt signaling pathway and its main function is to induce the classical Wnt signaling pathway, which is agonistic for promoting the proliferation of stem cells, inducing cell transformation and even the proliferation of tumor cells,^8^, ^9^ and also regulates apoptosis and cell proliferation in different cell types.^10^, ^11^, ^12^ It was found that activation of the Wnt signaling pathway induces proliferation and differentiation of cochlear precursor cells into hair cells,^13^ and that Notch, Wnt and SHH signaling pathways can jointly regulate and promote extensive proliferation and regeneration of mouse cochlear cells.^14^ Thus Wnt signaling pathway plays a protective and proliferation-promoting role in the development of mammalian inner ear auditory cells. Brown et al. found that Wnt1 and Wnt3a secreted from the dorsal neural tube are required for the expression of several key dorsal otic determinants and vestibular development was completely impaired in Wnt1^−/−^Wnt3a^−/−^ embryos.^15^ Hence, Wnt3a may play a significant role in development and injury repair process of auditory epithelium. However, whether Wnt3a has a regulatory role in the oxidative damage process of cochlear hair cells is rarely studied.

In this study, peroxynitrite was used as oxidative damage factor and the cochlear basal membrane was cultured in vitro to investigate the damage effect of peroxynitrite on hair cells. Accordingly, the aim of the present study was to preliminary explore the regulatory role of Wnt3a which regarded as an activator of the canonical Wnt signaling pathway in this process, in order to find new theoretical support for the pathogenesis and therapeutic targets of SNHL caused by hair cell damage.

## Methods

### Animals

C57BL/6 J healthy mice were purchased from Beijing Viton Lihua Laboratory Animal Technology Co., with the animal production license number: SCXK (Beijing) 2016−0006. The mice at Postnatal day 3 (P3) were taken for the experiment.

### Cochlear organotypic cultures

Postnatal C57 BL/6 J P3 suckling mice were decapitated after full body disinfection and the skulls were opened along midline of sagittal suture with ophthalmic straight knives and fine forceps. Temporal bones of two sides are cut and placed into sterile Hank’s Balanced Salt Solution on a flat ice pack. The cochlear capsule was removed by fine forceps to expose the membranous labyrinth under the dissecting microscope. The stria vascularis was peeled from the cochlear lateral wall. Organ of Corti and spiral ganglion samples were cut from their surrounding tissues. The entirety of each region from cochlear apex to base was isolated and spread on the slide as far as possible according to the physiological spiral structure. Whole basement membrane were then placed onto 10 mm coverslips pre-coated with CellTaK according to the rotation Angle of physiological state, and incubated in 48-well plate containing DMEM/F12 supplemented with 10% FBS and ampicillin (50 mg/mL) at 37 °C in a 5% CO_2_ atmosphere for 24 h.

### Peroxynitrite exposure and procedures

The cultured cochleae in vitro were randomly selected into three groups, including 100 μM peroxynitrite, l00 μM peroxynitrite +25 ng/mL Wnt3a, and the normal control group (PBS). Each experiment was performed in triplicate. Dose and administration schedules of peroxynitrite and agents used here were chosen based on our previous results.^16^ Peroxynitrite was purchased from Cayman, USA. Wnt3a was purchased from Sigma, USA.

### Immunohistochemical fluorescence staining

The cochlear basement membrane was fixed in 4% paraformaldehyde for 30 min to 1 h, permeabilized in PBS with 0.1% triton-X 100 (PBST) for 10 min at room temperature, blocked with 1% BSA in PBS for 1–1.5 h, incubated with primary mouse anti-myosin7a primary antibody (Abcam, USA. Diluted at 1:1000) and incubate at 4 °C for 16–18 h overnight, washed three times in PBS in the next day, and then incubated with the goat anti-mouse Alexa Flour-488 fluorescent for 1 h in dark at room temperature. Specimens were observed under a laser scanning confocal microscope (TSC SPE, LEICA) with a system offering 488-nm solid-state diode-pumped laser for excitation.

### Hair cell count

**Figure 1:**
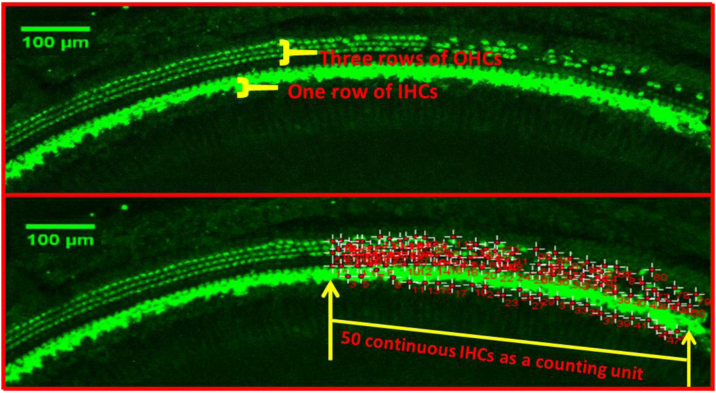
Quantitative evaluation of HCs with Myosin7a immunostaining. Take the total length of 50 continuous IHCs as a counting unit, count the number of corresponding OHCs for each segment.

Quantitative evaluation of HCs was performed after the immunostaining with Myosin7a. The basal, middle and apex turn of the basement membrane were transferred to the confocal immunofluorescence microscope to take pictures, respectively. 3 typical damaged areas of hair cell were taken at every turn under 10× microscope, take the total length of 50 continuous Inner Hair Cells (IHCs) as a counting unit under ImageJ software, count the number of corresponding Outer Hair Cells (OHCs) for each segment (as shown in Fig. 1).

### Transmission electron microscopy (TEM)

The cochlear basement membrane of each group was fixed in 2% glutaraldehyde in 0.1 M sodium cacodylate, washed with sodium dimethyl arsenate buffer at 4 °C, and fixed in 1% osmic acid for 1.5–2 h. The tissues were infiltrated in gradient concentration ethanol uranium acetate saturated solution and ethanol successively, embedded in Spurr’s epoxy resin via propylene oxide with propylene oxide and embedding solution at 1:1, and then immersed in pure embedding solution. The template tank was filled with embedding solution for embedding and ultra-thin section making. Ultrathin sections (90 nm) were cut on an Ultracut S microtome, mounted on 200-mesh Athene thin bar-grids, and contrasted with uranyl acetate and lead citrate. Grids were examined using a TEM.

### Statistical analysis

In the case of significance (*p* < 0.05), the data were tested using the unpaired (independent) *t*-test in GraphPad Prism 8.0 to determine which groups are significantly different from each other. Where appropriate, results were expressed as mean ± Standard Deviation (SD). The significance level was indicated by the number of asterisks (*p < 0.05, **p < 0.01, and ***p < 0.001, respectively).

## Results

### Morphological observation of hair cells

**Figure 2:**
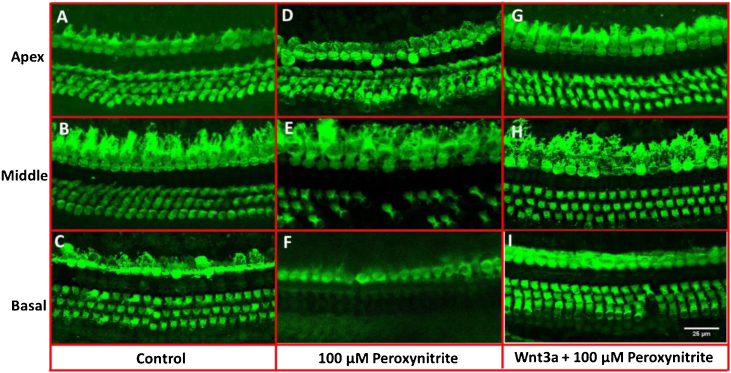
Immunostaining with Myosin7a for HCs. The IHCs and OHCs in the control group were arranged neatly in order with clear cell profile (A–C), whereas, in 100 μM peroxynitrite group, HCs were severely damaged in the basal and middle turn, especially the OHCs in the basal turn (D–F). In contrast, the arrangement of IHCs and OHCs in Wnt3a + peroxynitrite group was regular with few OHCs missing, the cell structure was clear (G–I).

In order to determine the possible protective effects of Wnt3a on the oxidative damaged auditory HCs, the cultured cochleae were treated with peroxynitrite and Wnt3a together at 100 μM for 24 h respectively in vitro, and then prepared for confocal microscope examination via immunostaining with Myosin7a antibody. The images showed that the one row of IHCs and the three rows of OHCs in the control group were arranged neatly in order with clear cell profile, with few or no HC loss being seen after being cultured for 48 h (Fig. 2A–C), whereas, after treatment with 100 μM peroxynitrite, HCs were severely damaged in the basal and middle turn, in detail, HCs were missing to varying degrees, especially the OHCs in the basal turn, and the arrangement pattern of OHCs became disordered, while the IHCs were not depleting obviously (Fig. 2D–F). In contrast, the arrangement of IHCs and OHCs in Wnt3a + peroxynitrite treatment group was regular, the cell structure was clear. HCs were significantly reduced with few OHCs missing was detected in individual areas (Fig. 2G–I). The results suggest that peroxynitrite can cause cochlea hair cells damage, and low concentration of Wnt3a can reduce the damage effects of peroxynitrite to the HCs morphologically.

### Wnt3a can improve the survival rate of HCs damaged by peroxynitrite

**Figure 3:**
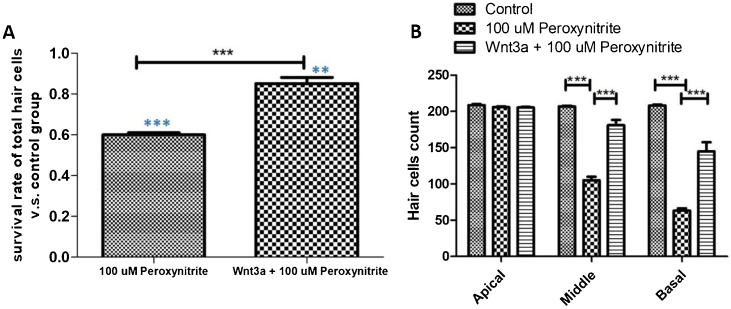
HCs count statistical analysis. (A) shows that the number of surviving HCs was significantly reduced in the 100 μM peroxynitrite group compared with the control group (*p* < 0.001, light blue***), while the survival rate of HCs in Wnt3a + peroxynitrite group was significantly higher compared with the peroxynitrite group (*p* < 0.001, black***). (B) shows that there was a significant difference in the reduction in the number of HCs in the middle and basal turn in the peroxynitrite-treated group compared with the normal control group (*p* < 0.001). There was no significant difference in the reduction in the number of HCs in the middle and basal turn in the Wnt3a + peroxynitrite treated group compared to normal controls (*p* = 0.055).

The results of immunofluorescence staining and HC count revealed that the number of surviving HCs was significantly reduced in the 100 μM peroxynitrite group compared with the control group. The reduction was mainly in the OHCs, with an overall survival rate of 59.85% ± 0.034%, while the survival rate of IHCs and OHCs in the Wnt3a + peroxynitrite treated group was 98.26%±0.018%, which was significantly higher compared with the peroxynitrite-treated group (*t* = 33.16, *p* < 0.001). There was no significant difference in the absence of HCs in the apex turn between the three groups. There was a significant difference in the reduction in the number of HCs in the middle and basal turn in the peroxynitrite-treated group compared with the normal control group (middle turn: 206 ± 3.62, 105 ± 15.33; *t* = 20.41, *p* < 0.001; basal turn: 208.2 ± 3.64, 62.8 ± 11.32, *t* = 37.26. *p* < 0.001). There was no significant difference in the reduction in the number of HCs in the middle and basal turn in the Wnt3a + peroxynitrite treated group compared to normal controls (middle turn: 206 ± 3.62, 201.7 ± 7.62; *t* = 1.875, *p* = 0.085; basal turn: 208.2 ± 3.64, 204.2 ± 4.83, *t* = 2.07, *p* = 0.055). There was also a significant difference in the number of HCs in the middle and basal turn in the Wnt3a + peroxynitrite treated group compared to the peroxynitrite group (middle turn: *t* = 17.86, *p* < 0.001; basal turn: *t* = 35.19, *p* < 0.001). Taken together, these results indicated that peroxynitrite caused HCs loss in a basal-apical gradient manner in the mouse cochlea, different HCs had different sensitivity to the same peroxynitrite insult and, more importantly, OHCs were more vulnerable to peroxynitrite injury than IHCs. Meanwhile, these data also showed that Wnt3a could protect HCs against the effect of peroxynitrite-induced HCs damage statistically (Fig. 3).

### Ultrastructural analysis via TEM

**Figure 4:**
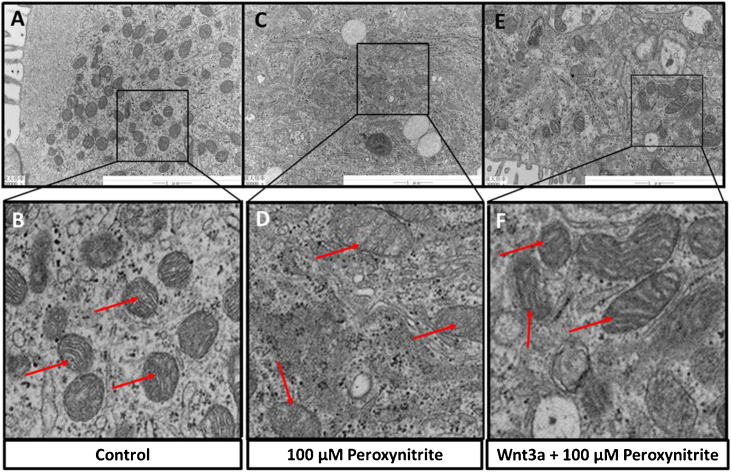
Ultrastructural analysis via TEM. TEM sections showed abundant mitochondria in the cytoplasm of HCs in the control group, with relatively uniform and clear mitochondrial cristae, respectively (A, B, red arrow). Peroxynitrite induced a dramatic decrease in the number of mitochondria and severely disrupted mitochondrial ultrastructure, with apparent swelling, mitochondrial cristae membrane fusion, and cytoplasmic vacuolation somewhere in HCs (C, D, red arrow). Wnt3a clearly diminished the disruption of mitochondrial structure and preserved a higher number of mitochondria, with irregular distribution, clear mitochondrial cristae, and mild swelling (E, F, red arrow).

The ultrastructural changes of HCs were observed by TEM sections, which showed abundant mitochondria in the cytoplasm of HCs in the control group, with relatively uniform and clear mitochondrial cristae, respectively (shown in Fig. 4A–B). While exposure to peroxynitrite induced a dramatic decrease in the number of mitochondria and severely disrupted mitochondrial ultrastructure, with apparent swelling, mitochondrial cristae membrane fusion, and cytoplasmic vacuolation somewhere in HCs (shown in Fig. 4C–D). Wnt3a clearly diminished the disruption of mitochondrial structure and preserved a higher number of mitochondria, with irregular distribution, clear mitochondrial cristae, and mild swelling (Fig. 4E–F). The above results suggest that peroxynitrite can cause oxidative damage to the mitochondria in HCs, which is closely related to energy metabolism with the destruction of cristae, and Wnt3a has a protective effect against this oxidative damage.

## Discussion

Peroxynitrite, the product of the reaction of nitric oxide with superoxide radical, is capable of oxidizing and nitrating a variety of biological targets including mitochondria and DNA,^17^, ^18^ and these modifications may be responsible for a number of pathological conditions and diseases.^19^, ^20^, ^21^ Studies showed that peroxynitrite is a crucial player in post-traumatic oxidative damage and a potent inducer of cell death.^22^, ^23^ Evidence indicate that most of the cytotoxicity attributed to NO is rather due to peroxynitrite, produced from the diffusion-controlled reaction between NO and the superoxide anion.^24^, ^25^ These reactions trigger cellular responses ranging from subtle modulations of cell signaling to overwhelming oxidative injury, committing cells to necrosis or apoptosis. In vivo, peroxynitrite generation represents a crucial pathogenic mechanism in conditions such as stroke, myocardial infarction, diabetes, chronic inflammatory diseases, and neurodegenerative disorders.^25^, ^26^, ^27^ As we known, hearing loss is closed related with oxidative stress induced by different otology diseases such as suppurative otitis media, age-related and noise-induced hearing loss.^28^, ^29^, ^30^ While there are few studies that use peroxynitrite as a direct damage factor in otology, occasionally in cochlear spiral ganglion cell damage,^31^, ^32^ and rare studies for peroxynitrite damage to HCs.^16^ In this study, we performed primary culture in vitro on C57BL/6 J mouse cochlear basement membrane with peroxynitrite, and found that peroxynitrite inducing HCs damage tended to increase from the apex turn to the basal turn, suggesting the HCs in the basal turn were the most sensitive to peroxynitrite damage. We also observed a decline in the number of mitochondria, cytoplasmic vacuolization, and reduction of mitochondrial cristae in HCs, and other ultrastructural manifestations of cellular oxidative damage via TEM. As Radi et al. found that peroxynitrite arising from extramitochondrial sites can reach mitochondria and cause oxidative damage and peroxynitrite promotes mitochondrial oxidative damage by primarily causing oxidation,^33^ our research found the similar results that peroxynitrite could induce mitochondria damage in HCs. All these results confirm the oxidative damage of peroxynitrite to HCs and provides a good tissue model for the study of SNHL caused by multiple oxidative injury factors.

Studies have found that antioxidants do have experimental and clinical practical value for oxidative stress induced hearing loss caused by various triggers. The role of antioxidants in prevention of Age-Related Hearing Loss (ARHL) has been reported, C57BL/6 mice fed with control diet or diet containing antioxidant compounds, ARHL was nearly completely prevented by antioxidant such as α-lipoic acid and coenzyme Q1.^34^ Of course, some of achievements demonstrate therapeutic potential, but a clinical application is still a long way off. A multicentre, randomised, open-label, phase 3 trial that enrolled participants at 38 participating Children's Oncology Group hospitals in the USA and Canada, found that, Sodium thiosulfate, as an antioxidant, could protect against cisplatin-induced hearing loss in children and is not associated with serious adverse events attributed to its use.^35^ Therefore, the application of antioxidants in hearing loss is indeed worth further research.

Adi Shruster et al. demonstrated that Wnt3a enhances neural regeneration after focal cerebral ischemia and has an ameliorating effect on neurological dysfunction.^36^ It has also been found that low concentrations of Wnt3a can act as a stimulus for HCs regeneration to induce the conversion of supporting cells or stromal cells in the ellipsoid capsule to HCs.^37^ Our experimental results showed that low concentrations (25 ng/mL) of Wnt3a significantly increased the survival rate of HCs damaged by peroxynitrite and significantly reduced the damage of mitochondria and mitochondrial cristae, which are closely related to energy metabolism, suggesting that Wnt3a has a protective effect on the oxidative damage of cochlear HCs induced by peroxynitrite. Thus, inferring that the classical Wnt signaling pathway may have a regulatory role in the oxidative damage of HCs, and this damage may be closely related to the alteration of mitochondria.

In this paper, we only conducted a preliminary and superficial study on the effect of Wnt3a on the oxidative damage of cochlear HCs. We will use this as a basis for more in-depth molecular studies to further explore the role of the classical Wnt signaling pathway in the oxidative damage of HCs and thus provide new theoretical support for the prevention and treatment of SNHL, which also has important practical significance for the prevention and treatment of SNHL. Hence, novel pharmacological strategies aimed at removing Wnt3a might represent powerful therapeutic tools in the future.

## Conclusion

We revealed the role of peroxynitrite and Wnt3a to mouse cochlear HCs in vitro for the first time via primary culture. The results indicated that peroxynitrite could cause oxidative damage to the cochlear HCs, and low concentrations of Wnt3a has a protective effect against this oxidative damage. Further studies are needed to unveil the deep mechanism of Wnt3a regulatory role in the oxidative damage of HCs.

## Ethical approval

This animal experimental research was approved by the Institutional Animal Care and Use Committee of the Shandong First Medical University, China and was performed in institutional Experimental Animals Research and Application Center in accordance with the accepted policy on the use of animals. Committee Name: The Laboratory Animals Local Ethics Committee of Shandong Provincial Hospital Affiliated to Shandong First Medical University. Permit number: (NO.2022-110).

## Funding

Natural Science Foundation of Shandong Province (Grant number: ZR2021MH369); 10.13039/501100001809National Natural Science Foundation of China (Grant number: 82201293).

## Conflicts of interest

The authors declare no conflicts of interest.
